# Retraction Note: Estimation of flood environmental effects using flood zone mapping techniques in Halilrood Kerman, Iran

**DOI:** 10.1186/s40201-015-0182-2

**Published:** 2015-04-17

**Authors:** Siamak Boudaghpour, Majid Bagheri, Zahra Bagheri

**Affiliations:** Department of Civil Engineering, K.N. Toosi University of Technology, Vanak Square, Tehran, Iran; Department and Faculty of Basic Sciences, PUK University, Kermanshah, Iran

This article [1] has been retracted by the Editor as it was published in error and has already been published elsewhere [2]. After the peer review process, the authors uploaded the wrong manuscript in error. Due to an oversight, the manuscript was subsequently accepted and published. The authors, Editor and BioMed Central apologise to all involved.
